# DAM: Hierarchical Adaptive Feature Selection Using Convolution Encoder Decoder Network for Strawberry Segmentation

**DOI:** 10.3389/fpls.2021.591333

**Published:** 2021-02-22

**Authors:** Talha Ilyas, Muhammad Umraiz, Abbas Khan, Hyongsuk Kim

**Affiliations:** ^1^Division of Electronic Engineering, Intelligent Robots Research Center, Jeonbuk National University, Jeonju, South Korea; ^2^Division of Electronic and Information Engineering, Jeonbuk National University, Jeonju, South Korea

**Keywords:** semantic segmentation, convolutional neural network, encoder-decoder architecture, fruit segmentation, channel attention, spatial attention, segmentation grad-cam, autonomous harvesting

## Abstract

Autonomous harvesters can be used for the timely cultivation of high-value crops such as strawberries, where the robots have the capability to identify ripe and unripe crops. However, the real-time segmentation of strawberries in an unbridled farming environment is a challenging task due to fruit occlusion by multiple trusses, stems, and leaves. In this work, we propose a possible solution by constructing a dynamic feature selection mechanism for convolutional neural networks (CNN). The proposed building block namely a dense attention module (DAM) controls the flow of information between the convolutional encoder and decoder. DAM enables hierarchical adaptive feature fusion by exploiting both inter-channel and intra-channel relationships and can be easily integrated into any existing CNN to obtain category-specific feature maps. We validate our attention module through extensive ablation experiments. In addition, a dataset is collected from different strawberry farms and divided into four classes corresponding to different maturity levels of fruits and one is devoted to background. Quantitative analysis of the proposed method showed a 4.1% and 2.32% increase in mean intersection over union, over existing state-of-the-art semantic segmentation models and other attention modules respectively, while simultaneously retaining a processing speed of 53 frames per second.

## Introduction

Since the evolution of deep convolutional neural networks (DCNNs) from neural networks (Krizhevsky et al., 2012), machine learning has shown unprecedented performance on a number of machine vision and pattern recognition tasks such as image classification (Krizhevsky et al., 2012; Simonyan and Zisserman, 2014; He et al., 2016; Szegedy et al., 2016; ur Rehman et al., 2018), object detection and localization (Ren et al., 2015; Redmon et al., 2016; He et al., 2017; Nizami et al., 2020), and semantic and instance segmentation (Long et al., 2015; Ronneberger et al., 2015; Badrinarayanan et al., 2017; Bolya et al., 2019). Recently, unsupervised algorithms are also gaining popularity (Epifanio and Soille, 2007; Zhao and Kit, 2011; Xia and Kulis, 2017; Ilyas et al., 2020) due to their certain advantages over supervised ones (Huang et al., 2017; Lin et al., 2017c; Ilyas et al., 2020). Moreover, deep learning has also demonstrated unparalleled performance in the field of bioinformatics and computational biology (Wahab et al., 2019, 2020; Park et al., 2020).

Where DCNNs have found several intuitive applications in various fields in our everyday lives, they are also being used in agriculture for autonomous harvesting and seeding. A lot of work has been done in literature in this regard, like crop and weed classification (Dyrmann et al., 2016, 2017; Grinblat et al., 2016; Kussul et al., 2017), plant detection (Mohanty et al., 2016; Khan et al., 2020), land cover classification (Ienco et al., 2017; Kussul et al., 2017), and crop disease identification (Fuentes et al., 2018). Just like any other machine vision task, the implementation of DCNNs in agriculture comes with its own set of problems. By the same token, the real time segmentation and detection of strawberries in an unconstrained farm environment is a challenging task, as strawberries usually grow in clusters and are occluded by leaves, branches, and other fruits. Due to different light intensities sometimes backgrounds and fruits have the same texture and color. These commonly occurring phenomena in farms makes the task more difficult and reduces the accuracy of DCNNs (Sa et al., 2016; Xiong et al., 2019).

Strawberries are some of the most highly valued crops as they give the best yield under sheltered environments, and thus have a very high production cost (Sa et al., 2016). The most crucial time for strawberry crop is harvesting time because the fruit becomes overripe quickly and if picking gets behind it effects the whole crop. Moreover, hiring skilled laborers in horticulture accounts for most of the cultivation cost. This crop also needs intensive post-harvest care (Guerrero et al., 2017). Because of all these expenses, horticulture industries in general are bound to have small profit margins. In some regions, labor cost makes up more than half of the total production cost, e.g., 60% in Norway (Xiong et al., 2019). Furthermore, there is a decline in interest of joining the agriculture industry among the new generation of workers (Adhikari et al., 2019). Under all these challenges the food industry must keep up with the demands of the ever-growing population.

To overcome such problems, one potential solution is autonomous harvesting as it can reduce labor cost to a minimum and increase the crop yield quality by timely harvesting. Due to outstanding performances of DCNNs in computer vision tasks, robotics and unmanned systems are now faster and more reliable than ever. Which in turn has allowed their adoption into many real-life applications like the detection of crop rows, weeds, and seeding beds in fields of maize and rice (Guerrero et al., 2017; Adhikari et al., 2019; Ma et al., 2019).

**FIGURE 1:**
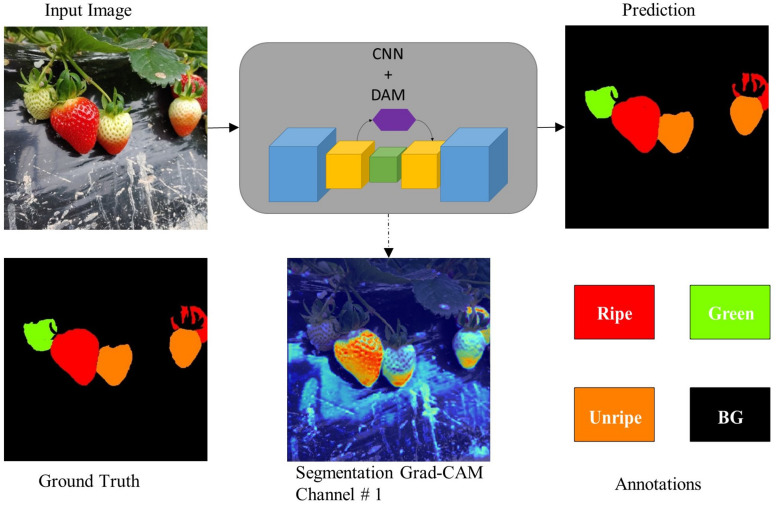
Overview of the proposed DCNN and the dataset with corresponding annotations. Seg-Grad-CAM represents the attention maps for ripe class (i.e., channel # 1).

**FIGURE 2:**
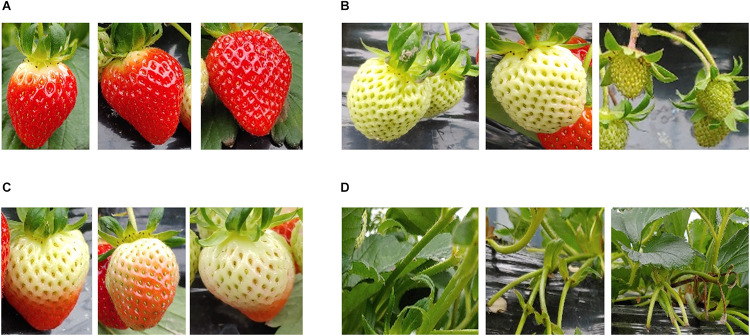
A few representative instances belonging to each class (stage of ripeness) in the strawberry segmentation (SS1K) dataset. **(A)** Ripe; edible quality, **(B)** green; not ready for harvest, **(C)** unripe; export quality, **(D)** background.

In this work, we proposed a DCNN named Straw-Net, to precisely segment and classify the fruits into specified classes in real time. In the case of strawberries, this is difficult to achieve because they usually grow in clusters and within the same cluster, and tend to have different sizes, shapes, and colors. In some cases, severe occlusion may also occur which renders the fruit almost invisible. By taking all these shortcomings into account, we designed an adaptive self-contained attention mechanism (i.e., dense attention module, DAM) for our network, which is capable of learning both channel and spatial interdependencies and can learn ‘what’ is important and ‘where’ to put more focus. We verify the efficacy of the proposed attention module quantitatively via benchmark metrics and qualitatively via modified Grad-CAM (Selvaraju et al., 2017). Grad-CAM is usually used for classification models, however, this paper extends its applicability to segmentation models. The dataset used in this paper is collected from different strawberry farms across the Republic of Korea under different lighting and weather conditions for better generalization of real-life scenarios. Our main contributions are listed below:

• We propose a single attention module (DAM) for both channel and spatial attention, and a parallel dilated convolution module (PDC) for aggregating multi-scale context.
• We validate the effectiveness of DAM and PDC by ample ablation experiments.
• We propose an optimal location for integrating our attention module in any existing network and compare results with other existing attention mechanisms.
• We propose a technique for visual interpretation of segmentation networks by modifying Grad-CAM.
• A new dataset for the semantic segmentation of strawberries is introduced, consisting of four classes depending on the ripeness level of fruit as shown in Figures 1, 2 (see section “Materials and Methods” for details).

## Related Work

### Autonomous Harvesting

A great deal of work has been done in the field of autonomous harvesting of fruits using various classical image analysis and pattern recognition techniques (Yamamoto et al., 2010; Feng et al., 2012; Ouyang et al., 2012; Qingchun et al., 2012; Zhang et al., 2012; Hayashi et al., 2013, 2014). One autonomous strawberry harvester (ASH) in particular AGROBOT SW6010 (Agrobot, 2020) has gained a lot of popularity. It uses morphological, color, and shape analysis for the identification and selection of strawberries to harvest, and uses 24 robotic arms to perform harvesting. FFRobotics (Kahani, 2017) have introduced the FFBot for harvesting apples, which needs a human supervisor to control and monitor the harvesting process. More recently, Hravest CROO (Harvest Croo, 2021) has introduced an ASH which divides the fruit picking into three steps, (a) grab the leaves, (b) 3D inspection of plant, and finally (c) pick the fruit. This simple robotic framework allows them to increase harvesting speed. Autonomous harvesters are also gaining popularity in other areas of precision agriculture like weeding. NAIO Technologies (Barthes, 2010) has introduced multiple autonomous weeding robots like OZ, TED, and DINO for easier vegetable weeding on large-scale farms.

The performance and accuracy of any autonomous harvester relies heavily on the perception system used and how the visual information obtained is being processed. An earlier era of vision-based harvesters used monocular devices to obtain 2D visual data, e.g., Grand d’Esnon et al. (1987) classified fruits based on their texture and geometry and Edan et al. (2000) used two monochromatic cameras to produce a stereo-like effect for better localization of melons. Similarly, Yamamoto et al. (2014) introduced a stationary strawberry picking mechanism which used three different monochromatic light sources for the coloration measurement and spectral analysis of fruits and leaves to precisely localize the fruit for picking. With this mechanism they achieved an effective yield rate of 67%. But these monochromatic vision systems were highly susceptible to light intensity changes. Later, some works used stereovision to obtain 3D map of fruits via triangulation (Sun et al., 2011). A predecessor of AGROBOT SW6010 used stereo RGB-D images for tomato harvesting (Buemi, 1995; Buemi et al., 1996). Using RGB-D images obtained via a Binocular-stereo vision camera, Ge et al. (2019) constructed a 3D point cloud to localize the pickable fruit. They used a Mask-RCNN as a backbone of their computer vision-based control to classify the strawberry into two classes, i.e., ripe and unripe. Following this pipeline they were able to improve the picking accuracy to 74%. Similarly, for recognizing clustered tomatoes and classifying them into overlapping and adhering regions, Xiang et al. (2014) used stereovision to obtain a depth-map, the reported accuracy for clustered tomato detection was 87.9%. Sensor calibration plays a vital role in the performance of stereovision systems. Recently, laser-based distance measuring systems (LiDAR) and spectral imaging are also doing wonders in precision agriculture. Zhang et al. (2015) combined computer vision with near-infrared structured lighting, and using a single multispectral camera was able to reconstruct the 3D surface of the apple for calyx and stem recognition. The results showed a 97.5% average accuracy.

These aforementioned ASHs rely heavily on classical mathematical algorithms; Ouyang et al. (2012) introduced a pipeline consisting of a series of image preprocessing and denoising techniques and then used the optimal thresholding (Otsu) algorithm for strawberry segmentation. Zhao et al. (2016) used a grayscale co-occurrence matrix to extract features of fruits from various color spaces. Whereas, different color spaces have different properties and their own application domain. Wei et al. (2014) used an OHTA color space, a modified version of the Otsu algorithm, to achieve an impressive detection accuracy of more than 95%. But it had a major drawback of not being able to detect green-colored fruits. Similarly, Qingchun et al. (2012) used a HSV color space to extract features for strawberry harvesting, results showed a 86% successful harvest rate. All the above-mentioned methods can detect fruits under controlled environments, but the detection accuracy drops when illumination changes. Moreover, depending on the variation in orientation, size, and shape of fruit, these methods require a lot of parameter tuning (Durand-Petiteville et al., 2017). In short, although several of the ASH prototypes have been developed to segment and classify fruits accurately in real time, their performance remains susceptible to unconstrained environments. This is where machine learning comes in.

Agricultural farms are unconstrained natural environments or semi-constrained at very best. Machine learning has found intuitive applications in many fields, because of its adaptive learning ability, like in healthcare (Ronneberger et al., 2015; Işın et al., 2016; Kauanova et al., 2017), autonomous driving (Fujiyoshi et al., 2019; Hofmarcher et al., 2019; Imai, 2019), and weed and crop detection (Grinblat et al., 2016; Mohanty et al., 2016; Dyrmann et al., 2017; Kussul et al., 2017; Fuentes et al., 2018). But very little work has been done in detecting fruits and classifying them according to their ripeness level. Lamb and Chuah (2018) used a single-stage detector SSD (Liu et al., 2016) to detect strawberries and attained a maximum average precision of 87.7%, but were not able to achieve real-time performance (see section “Real-Time Performance Barrier”) even after using various network compression techniques. Bargoti and Underwood (2017) proposed an image processing framework using a simple CNN and a multi-scale multi-layer perceptron (ms-MLP) to detect and count apples, with an F1-score of 85.8%. Their algorithm was a multi-stage setup which used watershed and circular Hough transform to detect the individual fruits. Hence falling short of real-time performance. Chen et al. (2019) used a faster region-based convolutional neural network (F-RCNN) (Ren et al., 2015) for predicting strawberry production rate using aerial farm images. Sa et al. (2016) presented an approach for fruit detection in field farms using an F-RCNN and showed its generalization to many different farm fields. Moreover, Yu et al. (2019) combined a Mask-RCNN with a feature pyramid network (FPN) for better feature extraction, to detect mature strawberries (one class) with a precision rate of 95%, but were not able to break the real-time performance barrier (see section “Real-Time Performance Barrier”). Whereas our proposed encoder-decoder based CNN is able to explicitly detect and classify fruits according to specified ripeness levels while still maintaining a processing speed of 53 fps on standard resolution images.

### Semantic Segmentation

Since the dawn of fully convolutional networks (FCNs) (Long et al., 2015) semantic segmentation has gained a lot of popularity. Following the main idea of embedding low contextual information in a progressive manner to preserve spatial and temporal information, a lot of encoder-decoder architecture has been introduced in literature. Deconv-Nets (Noh et al., 2015) introduced transposed convolution called deconvolution, for learning the upsampling process. SegNets (Badrinarayanan et al., 2017) introduced unpooling (i.e., inverse of pooling) to upsample the score maps in a gradual way. To remedy the loss of localization information by the subsequent downsampling of feature maps, U-net (Ronneberger et al., 2015) proposed skip-connections between the encoder and decoder to preserve spatial information. Further, the intermediate layers were exploited by RefineNet (Lin et al., 2017a) with skip-connections, which uses multipath refinement via different convolutional modules to get final predictions. Global Convolutional Network (Peng et al., 2017) tried to increase the receptive field by factorizing large kernels into smaller ones to get global contextual embeddings. PSP-Net (Zhao et al., 2017) used spatial pyramid pooling at different scales, and Deeplab (Chen et al., 2017) used atrous convolutions with different dilation rates for exploiting multi-scale information. Contrary to previous works that exploited intermediate layers by modifying identity skip-connections (Lin et al., 2017a; Peng et al., 2017) and those that use contextual multi-scale embedding for context gathering, our proposed network integrates the representational power of both of these types of networks to achieve better segmentation results.

### Dilated Separable Convolution

More recently, networks like Dilated ResNet (DRN) (Yu et al., 2017) used dilated convolutions (Yu and Koltun, 2015) to increase the valid receptive field size while still maintaining the same computational cost (i.e., number of parameters and FLOPs). Furthermore, Deeplab-v3+ (Chen et al., 2018) combined dilated convolution with depth-wise separable convolution (Chollet, 2017). By doing so they achieved a significant performance boost while keeping the model complexity to a minimum. These convolutions have been adopted by many recent algorithms (Jin et al., 2014; Wang et al., 2016; Howard et al., 2017; Zhang et al., 2018). In our network, we have also used the dilated separable convolution for better performance.

### Attention Mechanism

Attention plays a vital role in human perception (Rensink, 2000; Corbetta and Shulman, 2002). As a matter of fact, neurons present in the primary visual cortex of cats (Hubel and Wiesel, 1962) have inspired the construction of DCNNs (LeCun et al., 1989). Neurons in the human visual system do not process the whole semantic scene at once. Instead the neurons try to process the scenery in a sequence and they adaptively focus on only the salient features of the scenery in front of them (Woo et al., 2018).

Recent algorithms have also tried to equip DCNNs with such attention mechanisms to improve their performance (Lin et al., 2017b; Shen et al., 2018). More recently, Fu et al. (2019) proposed a self-attention mechanism for integrating local and global semantic features. Their mechanism consisted of two modules, one for position attention (PAM) and one for channel attention (CAM). Because of heavy matrix multiplications, both modules were far too computationally expensive. Whereas, squeeze and excite (SE) networks (Hu et al., 2018) recalibrated the feature maps depending upon their importance, while keeping the computational overhead to a minimum. Although in Hu et al. (2018), the authors implicitly refer to the SE module as an attention mechanism, this can be explicitly considered as one, as shown by Park et al. (2018) and Woo et al. (2018). Recently, a block attention module (BAM) (Park et al., 2018) and convolution block attention module (CBAM) (Woo et al., 2018) achieved a significant performance boost in an ImageNet-1K classification challenge by adding spatial attention to SE modules. These aforementioned modules also consisted of two separate blocks for generating channel and spatial attention. In contrast to these works, we extend the use of attention mechanisms to the segmentation task. Moreover, different from existing works, instead of using two separate blocks for channel and spatial attention, we propose one block for both tasks, to avoid computational overhead and reduce inference time. We propose a gating mechanism to control the flow of multi-scale information from different stages of the backbone network (encoder) to suitable upsampling stages of the decoder. By doing so, we are able to achieve better category specific attention masks. Our adaptive self-contained attention mechanism can learn both channel and spatial interdependencies and can dynamically emphasize or suppress features according to their importance. Detailed ablation experiments verify the effectiveness of our module (see section “Results and Discussion”).

## Materials and Methods

### Image Acquisition

**FIGURE 3:**
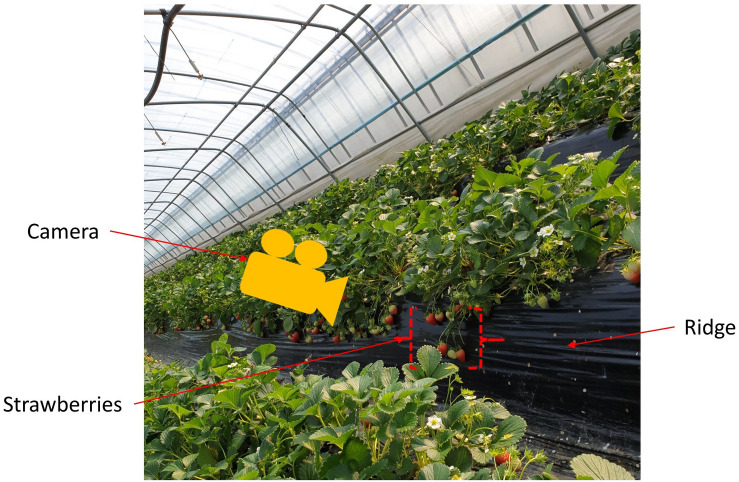
Data acquisition process.

Strawberry images were collected from several strawberry farms across Jeonju-si District, Jeollabuk-do, Republic of Korea during the growing season (2019). All the strawberry farms adopted a hedgerow planting system (Strawberry, 2020) as shown in Figure 3. The data acquisition was carried out at a distance of 40 cm, using a 24.1 MPx Canon EOS-200D-based platform with a CMOS sensor. We chose this distance so that the device could capture sufficiently large scenery for processing, and at this distance ASH would be able to perform suitable target searching and harvesting. During different time periods and under varying weather and lighting conditions, we acquired 1500 images. Images were stored in the JPEG format and all had a resolution of 6288 × 4056 pixels. We stored the data in high resolution to avoid being limited in available resolution at later processing stages.

### Dataset Construction and Annotation

We started with primary data filtering and removed the images which were blurred or contained no strawberry fruit at all. After the primary filtering step, we ended up with 1000 unlabeled images. Out of the total 1000 unlabeled images we randomly selected 750 images for training, 100 for validation, and 150 for testing.

**FIGURE 4:**
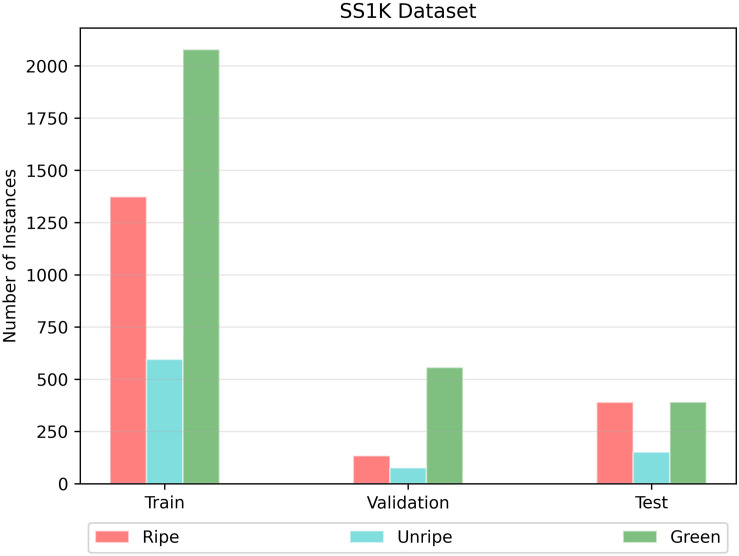
The number of instances of each class in the strawberry segmentation (SS1K) dataset.

Then with the help of experts in the strawberry harvesting field, we divided the strawberry fruits into four classes depending on the ripeness level. We labeled them as follows: (a) ripe; (edible quality) ready for harvesting, (b) green; not ready for harvesting, (c) unripe; (export quality) that can be harvested if the farm had to export the strawberries to far away destinations, and (d) background. Some representative instances belonging to each class (stage of ripeness) are shown in Figure 2. According to field experts and the Food and Agriculture Organization (FAO, 2020), a strawberry which is less than 70% matured should be considered as export quality. Because any more than that and there is a chance that the fruit may rot over long journeys. So, one might say that labeling the unripe class is somewhat intuitive. After deciding the ripeness level, we labeled the images as shown in Figure 1. There also exists a data imbalance between the classes, such that per batch there are a greater number of ripe and green strawberries than unripe ones, as shown in Figure 4. We will discuss this problem of data imbalance in the performance analysis (see section “Results and Discussion”). From this point onward for ease of notation we will call this strawberry segmentation dataset SS1K (1K for the total number of samples).

## Network Architecture

First, we will describe the backbone of our architecture, i.e., the encoder part and the blocks used within, and then we will discuss the design considerations for our attention module. Finally, we will describe the decoder design choice and how to integrate the attention module in any existing network.

### Encoder Design Considerations

**FIGURE 5:**
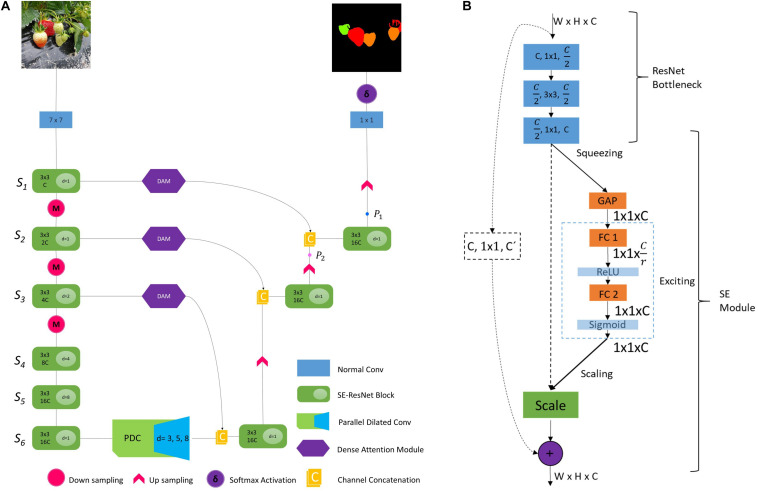
**(A)** Straw-Net, complete architecture. Here *S*_*i*_ represents different stages of network *i* ∈ {1,2,…6}. *c* represents the number of channels inside each SE-ResNet block (we set *c* = 16), and *d* is the dilation rate. For details on points P_1_ and P_2_ see Section “Ablation Study for DAM.” **(B)** SE-ResNet module; in the first three convolutional blocks, the first value represents the number of input feature maps, the second value represents the kernel size, and the third value represents the number of output channels. The dashed arrows represent the identity mapping. GAP, FC, and *r* represent global average pooling, densely connected layers, and reduction ratio, respectively.

A modified FCN for real-time segmentation of strawberry fruit, named Straw-Net is shown in Figure 5A. The encoder consists of SE-ResNet (Hu et al., 2018)-like blocks, with a few modifications. The SE-ResNet block consists of two parts, one being the ResNet bottleneck and the other being the SE-module as shown in Figure 5B. In the ResNet bottleneck, instead of using simple convolution, we decided to use the dilated separable convolution, which is a combination of dilated and depth-wise separable convolution (Chen et al., 2018). It allows the network designer to freely control the feature map’s size and filter’s effective receptive field (ERF), while significantly reducing the network computational cost. Depth-wise separable convolution disentangles the normal convolution into a depth-wise (or channel-wise) convolution followed by a point-wise convolution. This decomposition allows the DCNN to achieve better performance with much fewer parameters. In dilated convolutions, ERF can be easily changed by changing the dilation rate ‘*d*_*i*_’ (Yu et al., 2017), where normal convolution is a special case of dilated convolution with *d* = 1. Increasing the ERF at each stage of the network helps the convolutional filters to aggregate multi-scale contextual information more efficiently.

After processing the features by bottleneck layers, next these feature-maps are passed through the SE-module (Hu et al., 2018), shown in Figure 5B. SE-modules recalibrate the feature maps by obtaining their channel-wise statistics via global average pooling (GAP). The GAP outputs a vector of size *n*, where *n* is same as the number of filter channels. Then this vector is passed through a multi-layer perceptron (MLP) to obtain a weighing vector of size *n*. This vector is then used to adaptively emphasize or suppress the feature maps according to their importance. For more details about SE-modules, we refer interested readers to Hu et al. (2018). Moreover, skip-connection allows for uninterrupted gradient flow to the earlier layers for better training.

Data in raw images are mostly redundant so a large kernel size with high stride can be used to process the raw image and make it ready for deeper layers to process. Using a high stride also reduces the dimensions which will in turn reduce the computational overhead (Hasanpour et al., 2016; He et al., 2016). Keeping that in mind, firstly the image is passed through a normal convolution layer with 16 filters of size 7 × 7 and stride 2. Now this processed input is passed through the successive SE-ResNet blocks as shown in Figure 5A. Each convolutional layer in SE-ResNet is followed by a batch normalization (BN) and ReLu activation, unless explicitly stated.

The network backbone consists of six stages. All stages consist of two SE-ResNet blocks. Among those, the first three stages S_*i*∈ {1,2,3}_ are followed by subsequent pooling operations for reducing feature map size. In the next stages S_*i*∈ {4,5,6}_ we do not perform a pooling operation. Because, after using the stride = 2 in the first layer and the three subsequent pooling operations in the first three stages, the extracted feature map size is 16 times smaller than the input at the end of the encoder. Reducing it further will result in the loss of a lot of useful localization information, making the decoding process more difficult. In the first two stages, the dilation rate is set to *d* = 1, and in the next three stages, the dilation rate is doubled for every next stage, i.e., *d*_*i*∈ {2,4,8}_ for stages S_*i*∈ {3,4,5}_. The final stage S_6_ again has a dilation rate of *d_6_* = 1 to avoid the gridding artifact (Yu and Koltun, 2015).

### Parallel Dilated Convolution Module (PDC)

**FIGURE 6:**
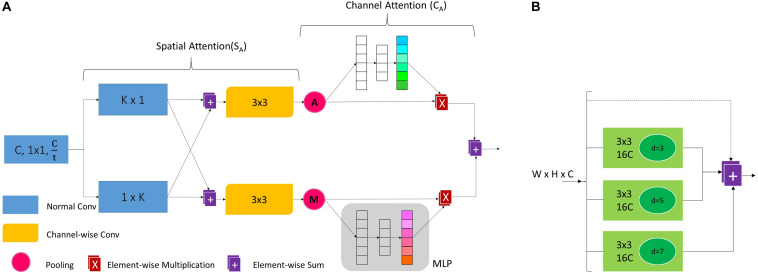
**(A)** Dense attention module, here *K* is kernel size and t is the reduction factor. **(B)** Parallel dilated convolution module, here *C* represent the no. of feature maps and *d* is the dilation rate.

We go deeper into the DCNNs, even though the deeper layers have a large theoretical receptive field (TRF) but their effective receptive field (ERF) is much smaller than the theoretical one as shown by Zhou et al. (2014). Information regarding global context plays a vital role in scene segmentation (Peng et al., 2017; Zhao et al., 2017). So, at the end of the encoder we probe the feature maps of the last stage (i.e., S_6_) for aggregating global and sub-region context by incorporating the PDC module shown in Figure 6B. PDC acts as a hierarchical global module prior to using dilated convolution at different dilation rates to extract global contextual information from S_6_’s feature maps at multiple scales. We perform detailed ablation experiments to show the effectiveness of PDC and compare it with other multi-scale feature aggregation modules of Zhao et al. (2017) and Chen et al. (2018) in Section “Ablation Study for (PDC).”

### Dense Attention Module (DAM)

To control the flow of information from encoder to decoder via skip-connections we incorporate the dense attention modules on skip-connections. We found that this is the best location to make the most out of these attention modules. These modules perform *‘feature surgery’* on the feature maps coming from the encoder which are rich in localization information. They help in efficient feature fusion between encoder feature maps (which focus on ‘where’ the target object is) and decoder feature maps (which focus on ‘what’ the target object is). The whole operation can be summarized as follows;

(1) $$F_{s_{i}}^{{}^{\prime \prime }}=D\,A\,M\,\left(F_{s_{i}}\right)$$

Given an input feature map *F_s_i__* ∈ ℝ^*W_S_i__*×*H_S_i__*×*C_s_i__*^ from stage *S*_*i*_ of the encoder, the DAM computes the refined feature map $F_{s_{i}}^{{}^{\prime \prime }}\in \,{\mathbb{R}}^{W^{{}^{\prime }}\times H^{{}^{\prime }}\times C^{{}^{\prime }}}$ to be concatenated with decoder feature maps. Usually, the low-level feature maps have a large number of channels (e.g., 128 or 256). So, DAM first reduces the number of channels of the corresponding low-level feature maps by a factor of *t* such that *F_s_i__* ∈ ℝ^*W_s_i__*×*H_s_i__*×*C*′^ where $C^{{}^{\prime }}=\frac{C_{s_{i}}}{t}$. To avoid the suppression of information in rich decoder feature maps by the low-level encoder feature maps, we set *t* = 4 in our experiments. The contextual information is aggregated using a large kernel size $f_{n}^{K\times K}$. To reduce the number of computations and inference time we decompose the one $f_{n}^{K\times K}$ filter into two parallel $f_{n}^{1\times K}$ and $f_{n}^{K\times 1}$filters. Here ‘*n*’ represents the normal convolutional filter and we set *K* = 7. Then the results of both these convolutions are added in their respective parallel branches as shown in Figure 6A. Next, we pass these feature maps through a depth (channel)-wise convolutional layer (in their respective branches) of filter size 3 × 3, i.e., $f_{c}^{3\times 3}$ where ‘*c*’ represents the depth-wise convolutional filter. In the depth-wise convolution, one filter convolves spatially on only one feature map making the output feature maps spatially enhanced as shown by Gao et al. (2018). So, the channel specific spatial attention for both branches is computed as,

(2) $$F_{s_{i}}^{S_{A}}=f_{c}^{3\times 3}\,\left(f_{n}^{1\times K}\,\left(F_{S_{i}}\right)+f_{n}^{K\times 1}\,\left(F_{S_{i}}\right)\right)$$

Here, the superscript *S*_*A*_ refers to spatial attention in the top (*S*_*t*_) and bottom (*S*_*m*_) branch. Next, this channel-specific spatial attention is recapitulated using both average and max pooling operations generating different feature descriptors.

(3) $$F_{S_{i}}^{a\,v\,g}=A\,v\,g\,P\,o\,o\,l\,\left(F_{s_{i}}^{s_{t}}\right)$$

(4) $$F_{S_{i}}^{m\,a\,x}=M\,a\,x\,P\,o\,o\,l\,\left(F_{s_{i}}^{s_{m}}\right)$$

Here, Fsip⁢o⁢o⁢l∈ℝW′×H′×C′′where *W*′ and *H*′ represent the pooled (average and max) width and height of the feature maps. Unlike previous work (Hu et al., 2018), we argue that instead of using only average pooling, exploiting both pooling operations to gather distinct global characteristics helps the module to infer distinct channel-wise attention in both branches independently. Exploiting both average and max pooling features greatly improves the network’s representational power (see section “Ablation Study for DAM”). After pooling, these 3D feature descriptors are passed through an MLP to obtain a 1D descriptor vector $F_{s_{i}}^{C_{A}}\in \,{\mathbb{R}}^{1\times 1\times C^{{}^{\prime }}}$, for obtaining channel attention *C*_A_ for both the top (*C*_t_) and bottom (*C*_m_) branch. MLP consists of one GAP layer for obtaining channel-wise statistical data and two neuron layers. These 1D vectors can now be used to scale their respective 3D feature maps according to their importance. In short, the channel attention is obtained as follows;

(5) $$F_{s_{i}}^{C_{t}}=\,M\,L\,P\,\left(F_{S_{i}}^{a\,v\,g}\right)$$

(6) $$F_{s_{i}}^{C_{m}}=\,M\,L\,P\,\left(F_{S_{i}}^{m\,a\,x}\right)$$

(7) $$F_{s_{i}}^{C_{t}}=\sigma \,\left[W_{2}\,\left(W_{1}\,\left(G\,A\,P\,\left(F_{S_{i}}^{a\,v\,g}\right)\right)+b_{1}\right)+b_{2}\right]$$

(8) $$F_{s_{i}}^{C_{m}}=\sigma \,\left[W_{2}\,\left(W_{1}\,\left(G\,A\,P\,\left(F_{S_{i}}^{m\,a\,x}\right)\right)+b_{1}\right)+b_{2}\right]$$

Here *W*_1_ ∈ ℝ^*C*′×*C*′/*r*^ and *b*_1_ ∈ ℝ^*C*′/*r*^ are the weights and biases of the hidden neuron layer while *W*_2_ ∈ ℝ^*C*′/*r*×*C*′^ and *b*_2_ ∈ ℝ*C*′ belong to the output neuron layer. Finally, the output of the module is now calculated as;

(9) $$F_{s_{i}}^{{}^{\prime \prime }}=\left(F_{s_{i}}^{C_{t}}⊗F_{S_{i}}^{a\,v\,g}\right)+\left(F_{s_{i}}^{C_{m}}⊗F_{S_{i}}^{m\,a\,x}\right)$$

Where, ⊗ denotes element-wise multiplication.

### Decoder Design Choices

We propose a simple yet effective decoder for our network as shown in Figure 5A. Our decoder bilinearly upsamples the feature map by a factor of 16 in subsequent steps. In the first step, the output of PDC is concatenated with the refined feature maps (i.e., output of DAM) of the third stage of the encoder then processed through a SE-ResNet block and finally upsampled by a factor of 2. The second and third steps also upsample the feature maps after concatenating and processing the feature maps in the same way. The only difference is that the second step upsamples by a factor of 2 while the third step upsamples by a factor of 4. This subsequent upsampling of feature maps after obtaining attention from DAM helps the network to further refine the segmentation results after each step. Lastly, the network’s output is obtained by performing a 1 × 1 convolution followed by Softmax activation.

### Implementation Details

Firstly, we resized all the images and segmentation masks to a 512 × 512 resolution without preserving the aspect ratio, to reduce training time and computational requirements. We also carried out extensive data augmentation during training to increase dataset size and to avoid overfitting. As for augmentation techniques used, we only selected those transformations which were suitable for segmentation problems and increased the network’s robustness. To be precise, we used random crop-and-resize, random mirroring along the vertical axis, random rotation, and lastly, random brightness and saturation distortion.

In the encoder, for the number of channels in each stage, we set *C* = 16. At each stage, the number of channels (*C*) and the dilation rate (*d*) were successively increased as shown in Figure 5A. For the SE-ResNet block (Figure 5B), following Hu et al. (2018), we set the reduction ratio to *r* = 8. In the PDC module for global context aggregation, we set dilation rate to *d* = {3,5,7}, respectively, for the three parallel branches as shown in Figure 6B. Regarding DAM, implementation details are provided in Section “Dense Attention Module (DAM).” For training, following Chen et al. (2018) and Fu et al. (2019), we employed an Adam optimizer along with poly learning rate policy where,

(10) $$l_{r\,\_\,n\,e\,w}=l_{r}*{\left(1-\frac{i\,t\,e\,r}{t\,o\,t\,a\,l\,\_\,i\,t\,e\,r}\right)}^{p\,o\,w\,e\,r}$$

Here, we set power = 0.9, *l*_*r*_ = 0.005 and we used weighted cross entropy as a loss function. We adopted dropout of 0.25 and set the mini batch_size = 4. The network is trained for 9K iterations.

## Results and Discussion

### Ablation Study for DAM

To evaluate the effectiveness of DAM we performed several experiments and the results are reported in Table 1. Our baseline consisted of a simple encoder and decoder as described in Section “Network Architecture” along with simple U-Net (Ronneberger et al., 2015)-like skip-connections. Baseline did not include DAM and PDC modules. It can be seen clearly from Table 1 that DAM significantly increases the mean IoU from 79.57% to 88.79%, with a slight increase in computational cost. Furthermore, the experiments also show that if we use different pooling operations AMP (i.e., average and max) in different branches then the network performs better as compared to the attention module with only one pooling (i.e., AP or MP). Ablation studies also show the effect of inclusion and exclusion of channel (*C*_*A*_) and spatial attention (*S*_A_) from DAM [see section “Dense Attention Module (DAM)”].

**TABLE 1:** Ablation studies on DAM.

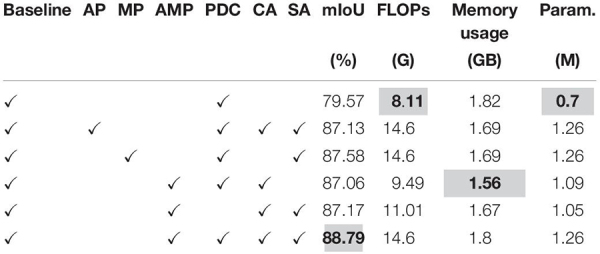

*Here AP and MP represent only using average or max pooling in both branches of DAM. AMP represents average and max pooling both being used in respective parallel branches of DAM. PDC is parallel dilated convolution module. *C*_*A*_ and *S*_*A*_ are channel and spatial attention sub-parts of DAM. Bold is used to highlight the best results.*

### Ablation Study for PDC

There are a number of modules available for multi-scale context aggregation for obtaining better feature representation from encoder feature maps, like the pyramid pooling module (PPM) of PSP-Net (Zhao et al., 2017). Furthermore, our PDC module is closer to the atrous spatial pyramid pooling modules namely ASPP (v2 and v3) introduced by Deeplab_v2 and Deeplab_v3 (Chen et al., 2017, 2018), respectively. We used a PDC module because it has smaller memory requirement, less floating-point operations (FLOPs), and number of parameters with almost identical performance. The results are summarized in Table 2.

**TABLE 2:** Ablation studies for PDC.

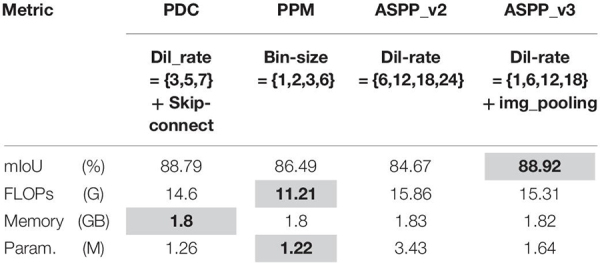

*PPM represents pyramid pooling module of Zhao et al. (2017) and ASPP_v2 and v3 represents the atrous spatial pyramid pooling modules of Chen et al. (2017) and Chen et al. (2018), respectively. Bold is used to highlight the best results.*

### DAM Visualization With Segmentation Grad-CAM

For the qualitative analysis, we apply the Grad-CAM (Selvaraju et al., 2017), to show the effects of DAM. Grad-CAM is a gradient-based visualization method, which tries to explain the reasoning behind the decisions made by the DCNNs. It was mainly proposed for classification networks. We propose a modified version of Grad-CAM to evaluate the results of the semantic segmentation model making it into Segmentation Grad-CAM (SGC). If ${\left\{A^{k}\right\}}_{k=1}^{K}$ represents the feature map of a selected layer with K feature maps then Grad-CAM calculates the heatmaps by taking the gradient of *y^c^* (logit for a given class) w.r.t to all *N* pixels (indexed by *u, v*), in all feature maps of ${\left\{A^{k}\right\}}_{k=1}^{K}$. But in the case of segmentation models, instead of *y^c^* (a single value), for each class we have $y_{i\,j}^{c}$ (a whole feature map). In this case, the gradients are computed by taking the mean of all *M* pixels (indexed by *i, j*) in the feature map of class ‘*c*.’ Finally, the weighing vector $\alpha_{k}^{c}$ is calculated as;

(11) $$\alpha_{k}^{c}=\frac{1}{N}\,\sum_{u,v}\left(\frac{\delta \,\frac{1}{M}\,\sum_{\left(i,j\right)}y_{i\,j}^{c}}{\delta \,A_{u,v}^{k}}\right)$$

The heatmaps are then generated by;

(12) $$L_{S\,G\,C}^{c}=R\,e\,L\,u\,\left(\sum_{k}\left(\alpha_{k}^{c}\,A^{k}\right)\right)$$

**FIGURE 7:**
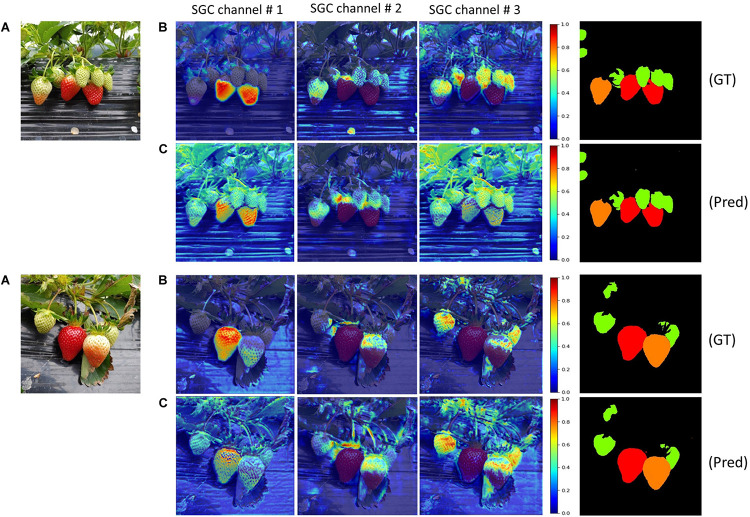
**(A)** Input images; **(B)** heatmaps at point P1; **(C)** heatmaps at point P2. GT represents ground truth and Pred. is the network’s prediction (best viewed in color).

Thus, SGC can produce heatmaps which explain the reasoning behind the grouping of individual pixels of the input image in one segmented region in the output. We display the activated attention maps of our network at two points in the decoder as shown in Figure 5A: firstly, after obtaining attention from DAM of stage S_1_ (i.e., point P_1_) and secondly after obtaining attention from DAM of stage S_2_ (i.e., point P_2_). The channel #s {1,2,3} correspond to the ripe, unripe, and green class of strawberry, respectively. It can be seen from Figure 7B that the heatmaps of all the classes at point P_1_ gets further refined and have clearer semantic meaning than those at point P_2_. Which shows the effectiveness of incorporating the DAM on skip-connections. For better visualization, all the heatmaps in Figure 7 have been rescaled to the same size.

### Comparison With State-of-the-Art Networks

**FIGURE 8:**
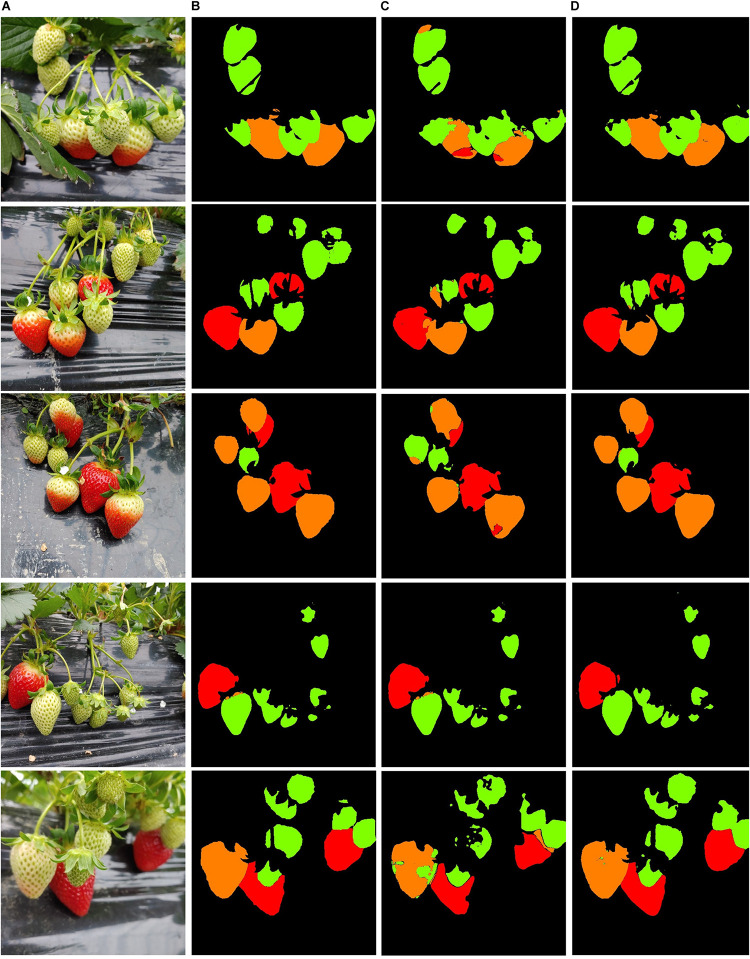
Visualization of some semantic segmentation results on the SS1K dataset. **(A)** Raw images. **(B)** Ground truth. **(C)** Deeplab v3+. **(D)** Straw-Net (best viewed in color).

In this sub-section, we compare the results of our network on the SS1K dataset with other existing state-of-the-art models in semantic segmentation. We evaluate all the models on different benchmark metrics and report the results in Table 3 and Figure 8 shows some visual semantic segmentation results. All the values reported in Table 3 are an average of 10 runs by default. Here IOR represents the input image to segmented output ratio. All the networks have a 1:1 ratio which means they output feature maps of the same size as the input, except DAN (Fu et al., 2019) and PSP-net (Zhao et al., 2017), their segmented output is eight times smaller than the input. Intersection over union value is averaged over all four classes. For precision and recall, the values are reported for each class separately and are calculated at a threshold of 0.75. It can be seen from Table 3 that our Straw-Net outperforms all other existing networks overall for real-time semantic segmentation of strawberry fruits. All the metrics including frames per second (fps) are calculated for 512 × 512 resolution images, on a single Nvidia Titan RTX-2080 GPU. From Table 3, it can be seen that our proposed network, even though incorporating an attention mechanism is much faster, requires less memory (GB) and less floating-point operations (FLOPs) as compared to other attention networks like DAN (Fu et al., 2019), BAM (Park et al., 2018), and CBAM (Woo et al., 2018). On the other hand, compared to other existing state-of-the-art segmentation models like Deeplab_v2 and Deeplab_v3 (Chen et al., 2017, 2018), our proposed approach is able to achieve a highest mean intersection over union (mIoU) value and comparable precision recall scores. The detailed architectures of all the networks used for comparison are provided as Supplementary Material.

**TABLE 3:** Comparison of results on SS1K dataset.

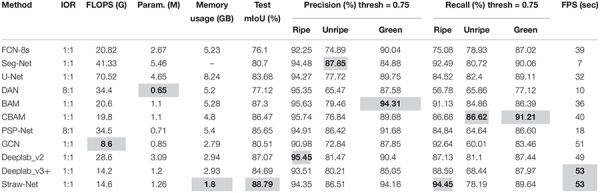

*IOR, input to output ratio of network; FPS, frames per second. For methods; bold is used to highlight the proposed method. For results; bold is used to highlight best results.*

### Further Analysis

**FIGURE 9:**
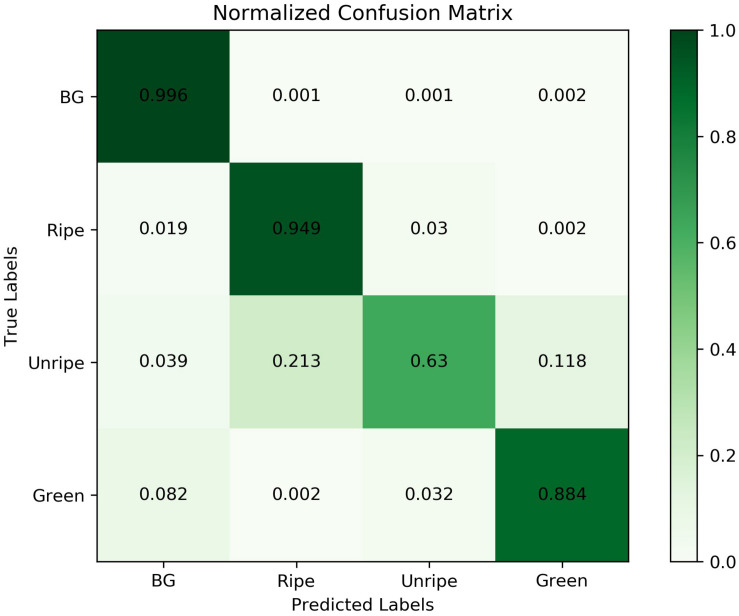
Confusion matrix for Straw-Net architecture for semantic segmentation of the SS1K dataset.

**FIGURE 10:**
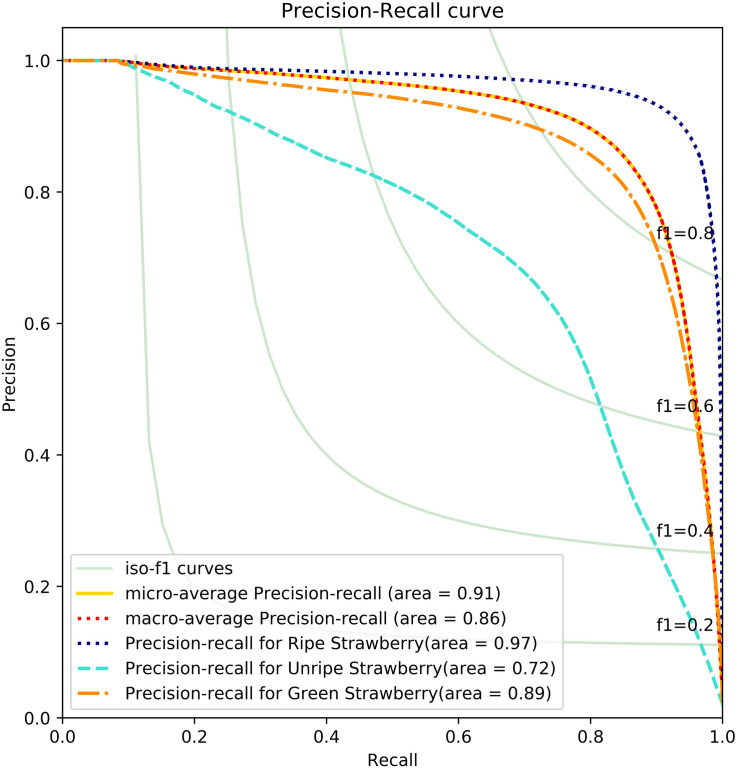
Precision recall curve obtained for Straw-Net architecture on the SS1K dataset (Best viewed in color).

To analyze the results further and to see which classes confuse the network resulting in lower performance, we plot a precision-recall (PR) curve and confusion matrix of the final segmentation results, as shown in Figures 9, 10. From the results we can analyze the networks performance visually and see which classes or features are highlighted by neurons. Moreover, it will also help us to take precautionary measures to avoid inter-class confusions. For instance, in Figure 9 the confusion matrix shows that the network is more confused between ripe and unripe strawberries rather than between unripe and green strawberries.

Another reason for this instability in confusion matrix is data imbalance, as shown in Figure 4. Because, there are fewer samples of unripe strawberries per batch as compared to the other two classes. We can analyze the effect of this imbalance from PR-Curves. We plot a PR-Curve for each class in Figure 10. PR-Curves represent a trade-off between precision and recall at different thresholds. The area under the precision-recall curve is usually denoted as AUC (i.e., area under the curve). A high value of AUC means high precision and recall. Whether we want high precision or high recall depends on the application domain. In Figure 10, ISO-F1 curves represents the lines in precision-recall space which have the same F1-values. We can see from Figure 10 that the AUC for unripe strawberries is much less than the ripe and green strawberries which in turn means that precision and recall values are also low for unripe strawberries. Moreover, the micro-average curve represents the mean of PR-Curves of all classes considering data imbalance. Whereas, the macro-average curve represents the mean PR-Curve without considering data imbalance.

#### Real-Time Performance Barrier

Neurons in the human visual system can interpret 10 to 12 fps and perceive them individually (Read and Meyer, 2000), whereas higher frame rates are perceived as motion. To reduce eye strain, the standard frame rate was set to be anywhere between 16 and 25 fps (Brown, 2014). Nowadays, all available video cameras have the minimum frame rate of 24 fps (Brunner, 2021).

For example, let us assume that a camera is generating 24 fps and sending those frames as an input to the proposed architecture, then the proposed algorithm should be able to process all those frames within a second to produce an output that is perceivable to the human eye. Therefore, if an algorithm can achieve a speed above this threshold (≥16 fps) it is said to have crossed the real-time barrier, where this limitation is mainly generated by the human perception system. In the case of ASH, if an algorithm has a processing speed of ≥24 fps it means that it will generate outputs (i.e., strawberry segments) after processing all the input frames. The processing speed of 53 fps was the maximum frame rate that was achieved during the experiments with the highest system configuration, i.e., RTX-2080 GPU and Core i9-9940X CPU as shown in Table 4. In contrast, for most sluggish situations, let us consider that a system can only process 3 fps (Laptop2 Core i5-8265 no GPU). In this case we might have to quantize our frames so that the network can process them before the next batch arrives. Thus, the statistical value of the output generated by a 53 fps system would be higher than the output generated by a 3 fps system.

**TABLE 4:** Comparison of different system configurations on network’s (Straw-Net) inference speed.

System	OS	CPU	Clock speed (GHz)	GPU (Nvidia)	FLOPS (Tera)	Power consumption (Watts)	FPS (sec)
*Server*	Linux 18.04	Core i9-9940X	3.3	RTX-2080	14.2	–	53
*Desktop PC*	Linux 16.04	Core i7-9700	3.0	GTX-1650	5.5	180 ∼ 300	28.9
*Desktop PC*	Linux 16.04	Core i7-9700	3.0	None	–	180 ∼ 300	13.8
**Portable devices**							
*Laptop0*	Windows 10	Core i7-10750	2.6	RTX-2070	6.6	170	40
*Laptop1*	Windows 10	Core i7-9750	2.59	None	–	70	6.32
*Laptop1*	Windows 10	Core i7-9750	2.59	GTX-1650	3.2	120	21.3
*Laptop2*	Windows 10	Core i5-8265	1.8	None	–	48	3.38
**Embedded systems**							
Nvidia Jetson TX2	Linux 16.04	ARM-Cortex A57	2.0	Pascal GPU	1.3	35	15.3

Our model is adaptive, easily scalable, has a small computational footprint of 14.6 GFLOPS (Table 3), and an even smaller memory footprint of 1.8 GB (Table 3). Therefore, it can be easily implemented on machines with low computational power like laptops with (40 and 21.3 FPS) or without (3.38 FPS) GPU or even on embedded systems like an NVIDIA Jetson TX2 board (15.3 FPS) without any loss in precision and accuracy. Therefore, any system configuration in ASH operating at the speed of ≥16 fps would overcome the real-time barrier and will be suitable for autonomous harvesting.

#### Effect of Input Resolution

To demonstrate the effect of change in resolution on the inference speed and precision of the network, we consider two more resolutions in addition to 512 × 512, i.e., 256 × 256 (low resolution) and 1024 × 1024 (high resolution). The results are reported in Table 5 (all experiments were performed under the same conditions).

**TABLE 5:** Performance comparison for input images of varying resolution.

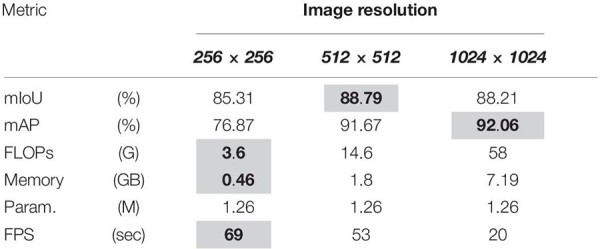

*GPU used for comparison is RTX-2080 (Table 4). Bold is used to highlight the best results.*

From the results we can see that if we reduce the resolution to 256 × 256 the computational complexity of the network is reduced considerably, and the speed is increased. Moreover, there is no significant decline in mIOU, but if we look at the AP, it is decreased by 14.8%. In contrast, if we analyze the case of high resolution (1024 × 1024), we can see that there is a little increase of about 0.58% in the network’s performance, but the computational complexity has exploded, and inference speed is now considerably slower than the 512 × 512 version. Therefore, we recommend using the 512 × 512 resolution.

## Conclusion

In this paper, a new dataset (i.e., SS1K) is introduced for the segmentation of strawberries into four classes depending upon the ripeness of the fruit (including a background class). The proposed segmentation network named Straw-Net improves the performance of ASHs in unconstrained and natural farming environments. Also, a real-time attention mechanism (DAM) is developed for integrating local and global semantic features efficiently. DAM controls the flow of information between the network’s encoder and decoder, enabling efficient feature fusion. Integrating adaptive feature fusion on skip-connections results in improved segmentation and classification ability of the network as shown by Segmentation Grad-CAM. The proposed attention mechanism can be integrated with any existing DCNN without any modification. By incorporating DAM in our baseline model, we achieved a significant performance boost while keeping the computational complexity to a minimum. Moreover, the effectiveness of DAM is verified by performing extensive ablation experiments. To verify the overall efficacy of the proposed approach, we compared the results with other attention mechanisms as well as with existing state-of-the-art segmentation models. Results demonstrated enhanced performance, i.e., improved mIoU, recall, and precision score with the proposed method on the strawberry segmentation problem. Our future work involves incorporating the proposed approach with ASH for deployment in strawberry farms.

## Data Availability Statement

The datasets generated for this study is available on request to the corresponding author.

## Author Contributions

TI designed the study, collected the data, performed the experiments, analyzed the data, and wrote the manuscript. MU and AK collected the data and performed the experiments. HK supervised and administered the overall project and reviewed and edited the writing. All authors contributed to the article and approved the submitted version.

## Conflict of Interest

The authors declare that the research was conducted in the absence of any commercial or financial relationships that could be construed as a potential conflict of interest.
