# Supplementary material for: DAM: Hierarchical Adaptive Feature Selection Using Convolution Encoder Decoder Network for Strawberry Segmentation
Source: Front Plant Sci. 2021 Feb 22;12:591333. doi: 10.3389/fpls.2021.591333 (PMC7937933; doi:10.3389/fpls.2021.591333)
Supplement: Supplementary file 1 [file Data_Sheet_1.PDF]

frontiers

Supplementary Material

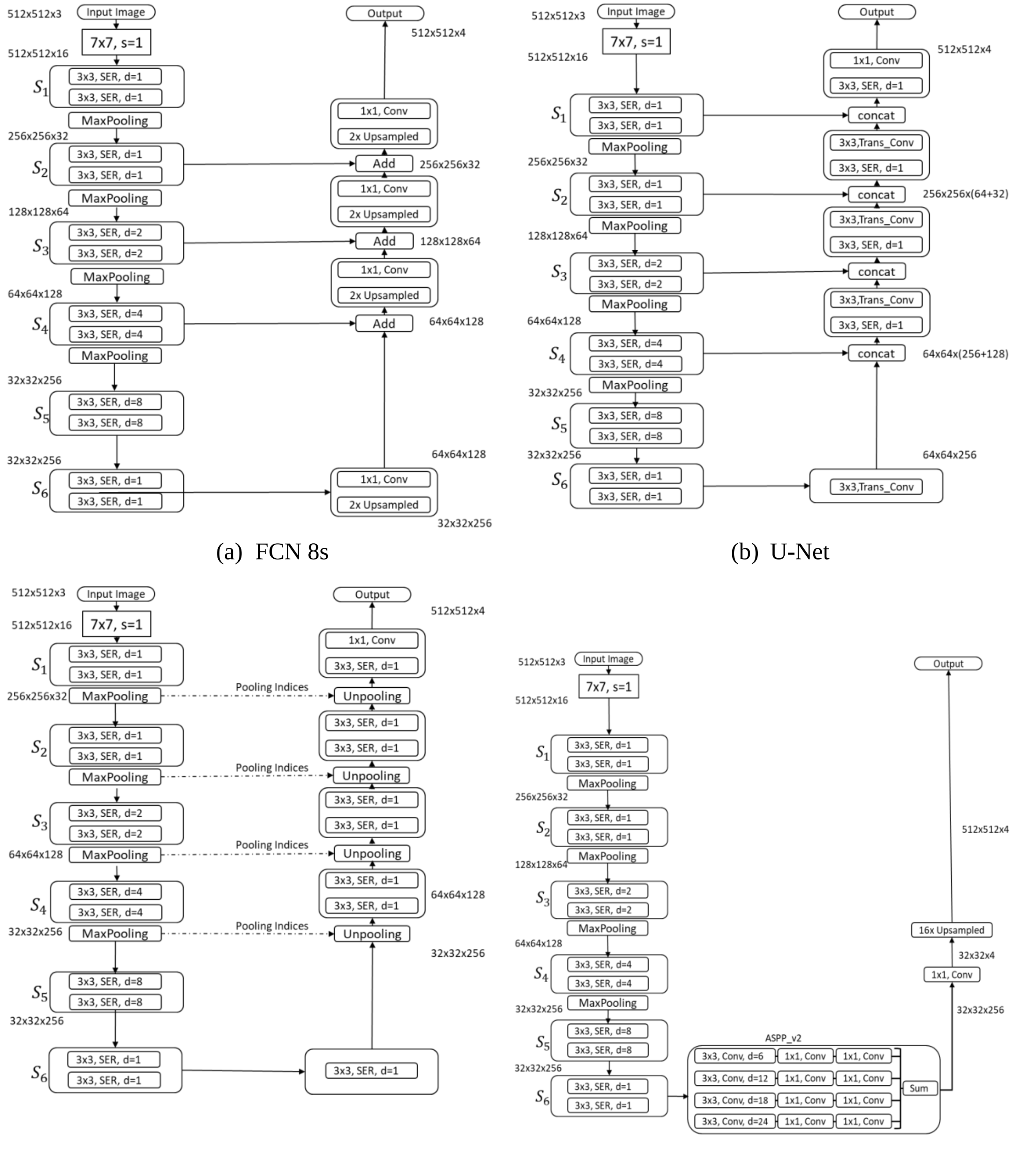

(a) FCN 8s

(b) U-Net

(c) Seg Net

(d) Deeplab v2

Supplementary Figure 1: Network architecture of (a)FCN 8s [8], (b) U-Net [9], (c) Seg Net [10], (d) Deeplabv2 [62]. Here SER represents SE-ResNet Block, '*s*' and '*d*' are stride and dilation rate. respectively.

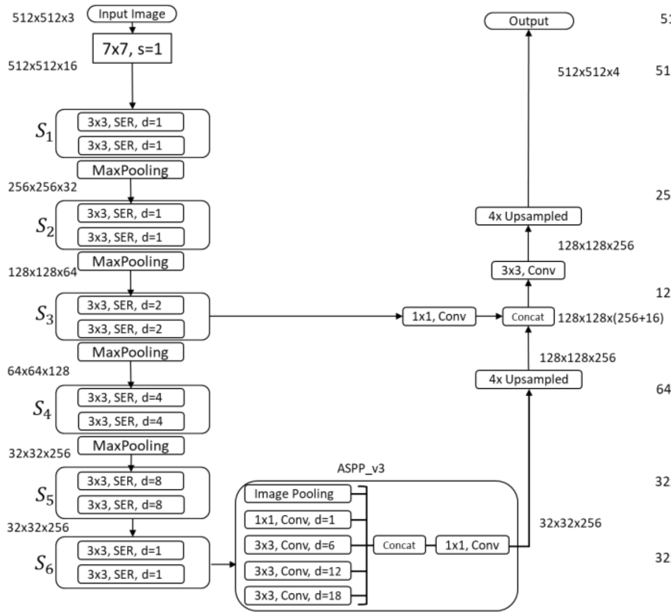

(a) Deeplab v3+

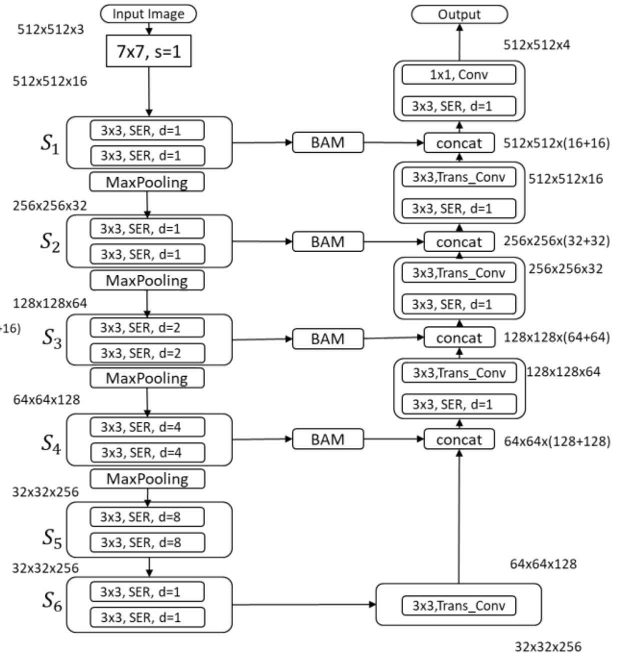

(b) BAM

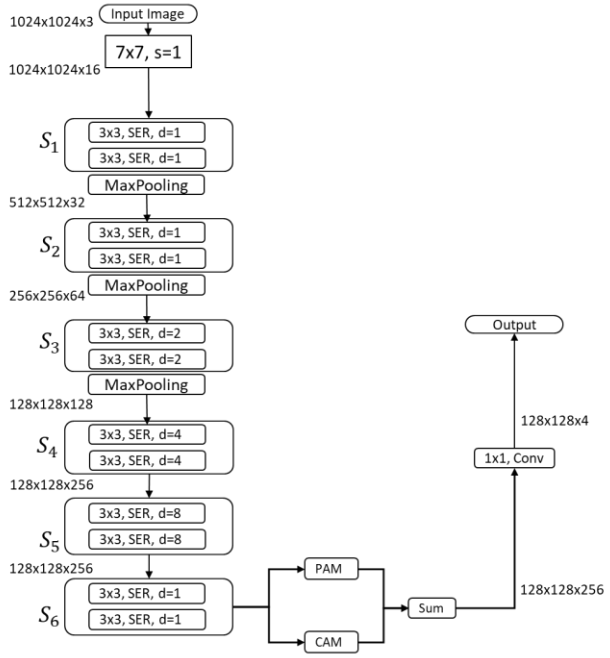

(c) DAN Net

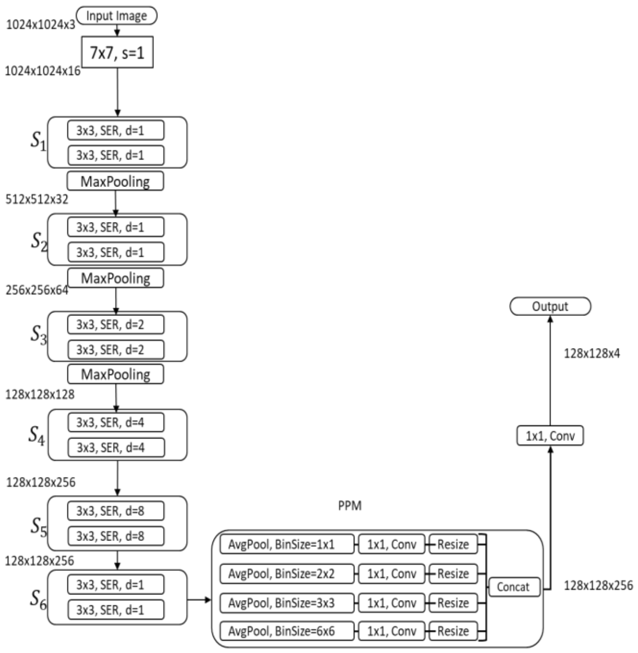

(d) PSP Net

Supplementary Figure 2: Network architecture of (a) Deeplabv3+ [60], (b) BAM [81], (c) DAN Net [80], (d) PSP Net [63]. Here SER represents SE-ResNet Block, '*s*' and '*d*' are stride and dilation respectively.

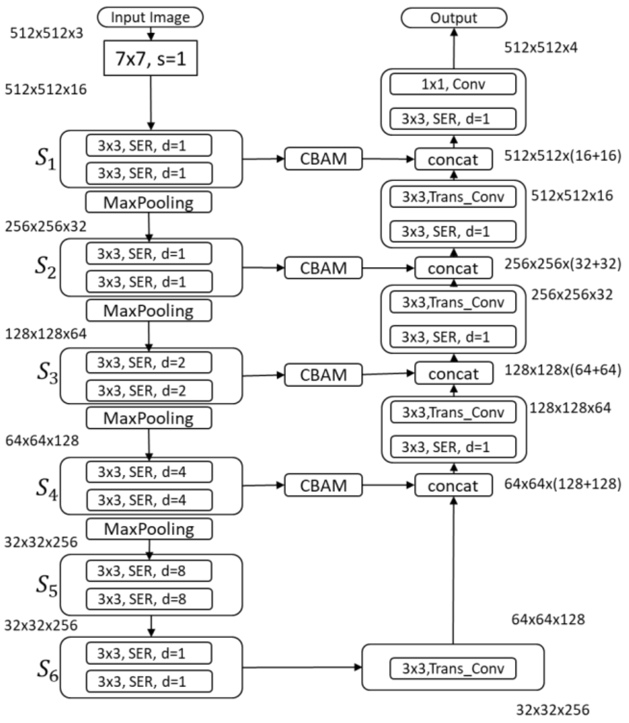

(a) CBAM

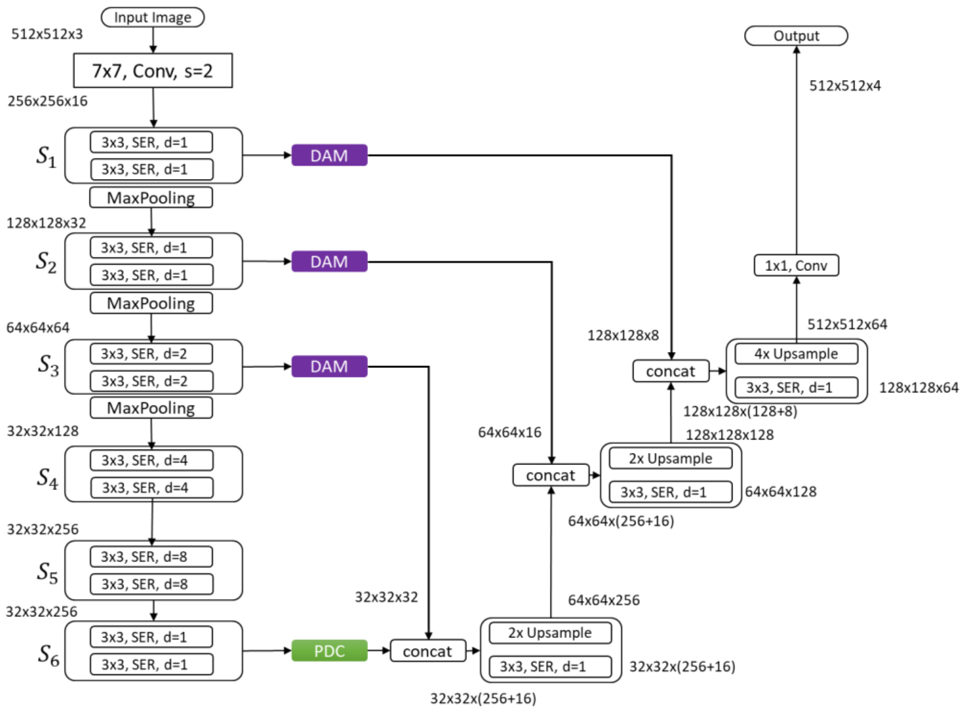

(b) SEED Net

Supplementary Figure 3: Network architecture of (a) CBAM [82], (b) Proposed Architecture. Here SER represents SE-ResNet Block, '*s*' and '*d*' are stride and dilation respectively.
